# Camel and bovine chymosin: the relationship between their structures and cheese-making properties

**DOI:** 10.1107/S0907444913003260

**Published:** 2013-04-19

**Authors:** Jesper Langholm Jensen, Anne Mølgaard, Jens-Christian Navarro Poulsen, Marianne Kirsten Harboe, Jens Bæk Simonsen, Andrea Maria Lorentzen, Karin Hjernø, Johannes M. van den Brink, Karsten Bruun Qvist, Sine Larsen

**Affiliations:** a Department of Chemistry, University of Copenhagen, Denmark; b Chr. Hansen A/S, Bøge Allé 10-12, DK-2970 Hørsholm, Denmark; c Nanobioscience, Department of Basic Sciences and Environment, University of Copenhagen, Denmark; d Institute of Biochemistry and Molecular Biology, University of Southern Denmark, Denmark

**Keywords:** aspartic peptidases, enzyme activity, substrate specificity, milk clotting, surface charge, domain flexibility

## Abstract

Bovine and camel chymosin are aspartic peptidases that are used industrially in cheese production. They cleave the Phe105-Met106 bond of the milk protein κ-casein, releasing its predominantly negatively charged C-terminus, which leads to the separation of the milk into curds and whey. Despite having 85% sequence identity, camel chymosin shows a 70% higher milk-clotting activity than bovine chymosin towards bovine milk. The activities, structures, thermal stabilities and glycosylation patterns of bovine and camel chymosin obtained by fermentation in *Aspergillus niger* have been examined. Different variants of the enzymes were isolated by hydrophobic interaction chromatography and showed variations in their glycosylation, N-terminal sequences and activities. Glycosylation at Asn291 and the loss of the first three residues of camel chymosin significantly decreased its activity. Thermal differential scanning calorimetry revealed a slightly higher thermal stability of camel chymosin compared with bovine chymosin. The crystal structure of a doubly glycosylated variant of camel chymosin was determined at a resolution of 1.6 Å and the crystal structure of unglycosylated bovine chymosin was redetermined at a slightly higher resolution (1.8 Å) than previously determined structures. Camel and bovine chymosin share the same overall fold, except for the antiparallel central β-sheet that connects the N-terminal and C-­terminal domains. In bovine chymosin the N-terminus forms one of the strands which is lacking in camel chymosin. This difference leads to an increase in the flexibility of the relative orientation of the two domains in the camel enzyme. Variations in the amino acids delineating the substrate-binding cleft suggest a greater flexibility in the ability to accommodate the substrate in camel chymosin. Both enzymes possess local positively charged patches on their surface that can play a role in interactions with the overall negatively charged C-terminus of κ-casein. Camel chymosin contains two additional positive patches that favour interaction with the substrate. The improved electrostatic interactions arising from variation in the surface charges and the greater malleability both in domain movements and substrate binding contribute to the better milk-clotting activity of camel chymosin towards bovine milk.

## Abstract 2

Analysis of the crystal structures of the two milk-clotting enzymes bovine and camel chymosin has revealed that the better milk-clotting activity towards bovine milk of camel chymosin compared with bovine chymosin is related to variations in their surface charges and their substrate-binding clefts.

## 1. Introduction   

Cheese production represents one of the earliest biotechnological applications of enzymes (Szecsi, 1992 ▶). Presumably, the first cheese production was merely an unexpected consequence of storing milk in bags made from the stomachs of ruminants (Tamime, 1993 ▶). The active ingredients in this process were identified as the proteolytic enzymes pepsin and chymosin, previously referred to as ‘rennet’ (Foltmann, 1966 ▶), in the early days of protein science (Fruton, 2002 ▶).

Both chymosin and pepsin belong to the pepsin-like family of aspartic peptidases (family A1 using the MEROPS classification; http://merops.sanger.ac.uk; Rawlings *et al.*, 2010 ▶) and their sequences are 55% identical. The inactive proenzymes contain an N-terminal prosegment of around 42 residues which is removed upon secretion into the acidic environment of the stomach, thereby leading to activation (Szecsi, 1992 ▶). Structural information is available for several members of the aspartic peptidase family. The crystal structures of porcine pepsin as a proenzyme (Sielecki *et al.*, 1991 ▶; Hartsuck *et al.*, 1992 ▶) and as an active enzyme (Cooper *et al.*, 1990 ▶; Sielecki *et al.*, 1990 ▶) are known. Structures have also been determined of native bovine chymosin (Gilliland *et al.*, 1990 ▶; Newman *et al.*, 1991 ▶), a mutant of bovine chymosin (Strop *et al.*, 1990 ▶) and an inhibitor complex (Groves *et al.*, 1998 ▶).

Pepsin and chymosin display the same overall structure, which is mainly comprised of β-sheets forming two similarly folded barrel domains. Structural comparisons have revealed repeating elements between and within each domain, which suggests gene duplications (Tang *et al.*, 1978 ▶). The substrate-binding cleft and active site are located at the interface of the two domains. An antiparallel β-sheet referred to as the central sheet (Šali *et al.*, 1992 ▶) connects the two domains and constitutes an independent structural element. The active site contains an activated water molecule held in position by two Asp residues: one from each domain (Cooper *et al.*, 1990 ▶; Gilliland *et al.*, 1990 ▶; Sielecki *et al.*, 1990 ▶; Newman *et al.*, 1991 ▶). The positions of the two catalytic Asp residues are secured through hydrogen-bond interactions with adjacent residues, forming an interdomain network referred to as the ‘fireman’s grip’ (Pearl & Blundell, 1984 ▶; Newman *et al.*, 1991 ▶). The catalytic mechanism proposed by Suguna *et al.* (1987 ▶) and James *et al.* (1992 ▶) involves a series of proton transfers triggered by nucleophilic attack of the water molecule between the two Asp residues. This proposed mechanism has recently been supported by neutron diffraction studies (Coates *et al.*, 2001 ▶, 2008 ▶).

The topology of the substrate-binding cleft can be described using the notation of Schechter & Berger (1967 ▶). The binding cleft can be divided into subsites (S), each occupied by one residue (P) of the substrate. These subsites and corresponding substrate residues are numbered counting away from the active site towards the N-terminus (S1 and P1, S2 and P2 *etc.*) and the C-terminus (S1′ and P1′, S2′ and P2′ *etc.*) of the substrate (*e.g.* residue P2′ binds in subsite S2′). The peptide bond between residues P1 and P1′ that is hydrolysed by the enzyme is referred to as the scissile bond. The substrate specificity of the enzyme depends on the properties (shape, charge *etc.*) of the subsites, which are therefore often referred to as ‘specificity pockets’. The aspartic peptidases are characterized by their large hydrophobic S1 and S1′ pockets and have a preference for substrates with large hydrophobic residues at P1 and P1′ (Kay & Dunn, 1992 ▶). Part of the binding cleft is made up by a β-hairpin flap comprised of residues 69–­79 (chymosin numbering), which was found to display a very large mobility in bovine chymosin. In the structure with bound inhibitor (Groves *et al.*, 1998 ▶) the flap was in the same conformation as in other peptidases, *e.g.* porcine pepsin (Cooper *et al.*, 1990 ▶; Sielecki *et al.*, 1990 ▶). However, in the native structure the flap adopted a different position with the side chain of Tyr77 blocking access to the substrate-binding cleft (Gilliland *et al.*, 1990 ▶; Newman *et al.*, 1991 ▶). It has been suggested that this self-inhibitory behaviour of bovine chymosin contributes to its increased specificity (Andreeva *et al.*, 1992 ▶), and kinetic studies have indicated that a *His-Pro* cluster in bovine κ-casein (all κ-casein residues are written in italics) acts as an allosteric regulator that converts chymosin into its active form (Gustchina *et al.*, 1996 ▶).

The clotting of milk is initiated by removing the hydrophilic and predominantly negatively charged C-terminus (Supplementary Fig. S4^1^) of κ-casein, the milk protein that forms part of the outer layer of the casein micelles. More specifically, the C-­terminus is removed by hydrolysing the *Phe105-Met106* bond of κ-casein or nearby bonds (Fox & McSweeney, 1998 ▶). This causes exposure of the hydrophobic core of the casein micelles, thereby leading to aggregation, gel formation and phase separation of the milk into curds and whey.

The dairy industry characterizes rennet enzymes using two properties. The first is the milk-clotting activity (*C*) expressed in International Milk-Clotting Units (IMCU). It is determined by a standard method (International Dairy Federation, 2007 ▶) that describes the ability to aggregate milk by cleaving the *Phe105-Met106* bond or nearby bonds of κ-casein. The second property is the general proteolytic activity (*P*), which is the ability to cleave any bond in casein (Kappeler *et al.*, 2006 ▶). The ratio between the two properties, the *C*/*P* ratio, captures the essential quality of a milk-clotting enzyme. The higher the value the better the rennet, and in this regard chymosin is superior to all other known rennet enzymes (Foltmann, 1992 ▶).

The *C*/*P* ratio of bovine chymosin towards bovine milk is higher than those of the chymosins from lamb, pig, cat and seal (Foltmann, 1970 ▶). However, camel chymosin produced in *Aspergillus niger* shows a sevenfold higher *C*/*P* ratio than bovine chymosin (70% higher clotting activity and only 25% of the general proteolytic activity; Kappeler *et al.*, 2006 ▶). Bovine and camel chymosin both consist of 323 residues and display high sequence identity (85%; Supplementary Fig. S4). In contrast, bovine chymosin displays a very low milk-clotting activity towards camel milk (Farah & Bachmann, 1987 ▶; Kappeler *et al.*, 2006 ▶).

The commercial bovine and camel chymosin products used in this investigation originate from production in *A. niger* (Harboe, 1992 ▶; Kappeler *et al.*, 2006 ▶). Natural bovine chymosin is found in two isoforms, A and B, which differ at residue 244, which is an Asp in the A form and a Gly in the B form (Foltmann, 1966 ▶). It is the B form that is used in the investigations described in this paper, as it is commercially available in large quantities at high purity; it is referred to as ‘bovine chymosin’. *A. niger* is known to glycosylate proteins at the N^δ2^ atom of an Asn found in the sequence Asn-*X*-Thr/Ser, where *X* cannot be Glu or Pro. The sites with Thr are found to have a higher degree of glycosylation than those with Ser (Harboe, 1998 ▶). These will be referred to as ‘more favoured’ (Thr) and ‘less favoured’ (Ser) sites. Bovine chymosin contains two less favoured glycosylation sites at Asn252 and Asn291 (Supplementary Fig. S4). Approximately 10% of the bovine chymosin produced by fermentation is glycosylated (unpublished data from Chr. Hansen A/S), but the distribution between the sites is unknown. Camel chymosin possesses two glycosylation sites: a favoured site at Asn100 and a less favoured site at Asn291. The extent of glycosylation of *A. niger* fermented camel chymosin has not previously been investigated.

The primary aim of the research presented here is to provide a structural understanding of why camel chymosin possesses a higher milk-clotting activity towards bovine milk than bovine chymosin. We report the separation and characterization of the variants of camel chymosin obtained from expression in *A. niger* and the crystal structure of one of the variants of camel chymosin to 1.6 Å resolution. The structure of bovine chymosin was determined more than 20 years ago (Gilliland *et al.*, 1990 ▶; Newman *et al.*, 1991 ▶; Strop *et al.*, 1990 ▶), and as no structure factors were available for these structures the structure of bovine chymosin has been redetermined benefitting from the improved methods of macromolecular crystallography. Based on X-ray synchrotron-radiation data, the structure of bovine chymosin has been redetermined to 1.8 Å resolution. These structures form the basis for detailed structural comparison that has identified structural differences that can explain the better performance of camel chymosin in terms of substrate recognition and action on κ-casein.

## 2. Methods   

### 2.1. Reagents and proteins   

Buffers were prepared using analytical grade chemicals followed by sterile filtration. The buffers used for fast protein liquid chromatography (FPLC) were degassed by ultrasonication at 42 kHz for 10 min. Transfers to new buffers were made using Econo-Pac 10DG columns. The bovine and camel chymosins were the commercial products Chy-MAX and Chy-MAX M, respectively, provided by Chr. Hansen A/S.

Bovine chymosin can be obtained by expression of the vector pGAMpR (Ward *et al.*, 1990 ▶) in the strain *A. niger* var. *awamori* GC1HF1-3;dgr246. This strain has been heavily modified for heterologous protein expression, for example by deletion of the gene coding for aspergillopepsin A in order to limit protein degradation (Berka *et al.*, 1991 ▶). Camel chymosin can be obtained by expression in the same strain of a modified vector, pGAMpR-C, that contains the camel chymosin gene (Kappeler *et al.*, 2006 ▶).

### 2.2. Separation of variants   

**Figure 1:**
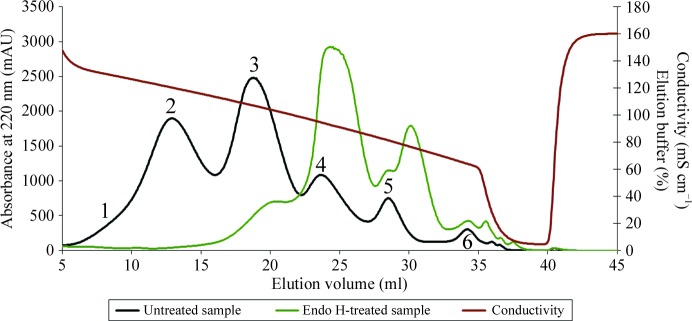
Representative chromatograms showing the FPLC separation of commercial camel chymosin using a salt gradient on a hydrophobic column. The black chromatogram shows the separated variant products numbered 1–6. The green chromatogram shows the separation of the product pretreated with endoglycosidase H.

The variants of bovine and camel chymosin were separated by hydrophobic interaction chromatography using an ÄKTApurifier 900 FPLC system. The commercial products were suspended in 12%(*w*/*v*) NaCl. The separation of camel chymosin variants was carried out by adding sodium sulfate to the commercial product to a final concentration of 0.5 *M*. The binding buffer was 12%(*w*/*v*) NaCl, 0.5 *M* sodium sulfate, 50 m*M* NaH_2_PO_4_ pH 6.5 and the elution buffer was 50 m*M* NaH_2_PO_4_ pH 6.5. Camel chymosin variants were separated on a Phenyl Superose column from Pharmacia. The separated camel chymosin variants 2 and 3 contained traces of the neighbouring variants (Fig. 1 ▶); hence, they were separated once more following the same procedure but adding salts to the sample to match the binding buffer. The variants were transferred to 50 m*M* bis-tris buffer pH 6.0 containing 0.05%(*w*/*v*) sodium azide.

Bovine chymosin variants were separated following the same procedure but using a concentration of 0.4 *M* sodium sulfate in the sample and binding buffers. The variants were transferred to 50 m*M* NaH_2_PO_4_ pH 6.0 containing 0.05%(*w*/*v*) sodium azide.

### 2.3. Deglycosylation   

Bovine and camel chymosin (0.9 and 2.1 mg ml^−1^, respectively) were deglycosylated with endoglycosidase H (Sigma, catalogue No. A0810). 25 milliunits was added per millilitre of sample, which was incubated at 278 K for 4 d. Separation of the deglycosylated samples followed the same procedure as described above for the commercial products.

### 2.4. Mass spectrometry   

The protein mass was measured using a Voyager Elite MALDI TOF mass spectrometer (Applied Biosystems Inc., Framingham, Massachusetts, USA) operated in linear positive-ion mode. The separated variants were desalted and concentrated on 50R1 microcolumns (Gobom *et al.*, 1999 ▶), subsequently eluted and deposited on a stainless-steel MALDI target with a matrix solution consisting of 20 mg ml^−1^ sinapinic acid in 70% acetonitrile and 0.1% trifluoroacetic acid. The target spots were pretreated with 0.5 µl sinapinic acid in acetone (20 mg ml^−1^). Samples were analysed in the mass range 3–50 kDa. The data were baseline-corrected and noise-filtered.

### 2.5. N-terminal sequencing   

An ABI 494 protein sequencer equipped with an ABI 140A microbore HPLC system (Applied Biosystems, Foster City, California, USA) was employed for N-terminal sequencing using sequencing-grade chemicals from Fluka.

### 2.6. Determination of glycosylation types and sites   

Chymosin samples were digested with trypsin (Promega; modified, sequencing grade) and Asp-N (Calbiochem; excision grade) (Højrup, 2009 ▶). 2 µg enzyme was added per 100 µg protein. After lyophilization approximately 2 µg of the sample was redissolved in 10 µl 80% acetonitrile and 2% formic acid and purified on a Polyhydroxyethyl A (PolyLC) or a hydrophilic interaction liquid chromatography (HILIC) microcolumn (Gobom *et al.*, 1999 ▶; Thaysen-Andersen *et al.*, 2007 ▶). The samples were eluted onto a target plate and mixed with 0.5 µl matrix solution as described by Thaysen-Andersen *et al.* (2007 ▶). Mass spectra (full scan and daughter-ion scans) were recorded using a 4800 Plus MALDI TOF/TOF (AB Sciex) mass spectrometer operated in reflector positive-ion mode. The acceleration voltage was 20 kV. Depending on the sample analysed, the laser intensity and the number of laser shots were varied to optimize the spectral appearance. The mass range was set to 700–8000 Da. For all MS/MS data air was used as the collision gas.

### 2.7. Assay for enzymatic activity   

The milk-clotting activity of the enzymes was determined using the standard milk-clotting assay (International Dairy Federation, 2007 ▶), which gives the activity in International Milk-Clotting Units (IMCU) by measuring the time required to achieve clotting of standardized skimmed milk compared with a standard enzyme sample. Using this method, the milk-clotting activities of the bovine and camel chymosin variants and the camel chymosin crystals resuspended in 50 m*M* bis-tris buffer pH 6.0 were determined.

### 2.8. Thermal stability measurements   

The melting temperatures (*T*_m_) were determined by differential scanning calorimetry (DSC) using a VP-DSC microcalorimeter (MicroCal). As the pH of bis-tris buffer varies significantly with temperature, all samples were transferred into 50 m*M* NaH_2_PO_4_ buffer pH 6.0 containing 0.05%(*w*/*v*) sodium azide. The samples were degassed for 5 min prior to measurements.

The samples were scanned at a rate of 1 K min^−1^. An initial scan of the two commercial products was made in the range 293–383 K. A single transition point was found at about 333 K. This information was used to set the temperature range to 313–343 K for the separated variants with concentrations of 2.8–20.0 µ*M*. At higher concentrations an exothermic contribution interfered with the signal for camel chymosin. At protein concentrations below 20 µ*M* there was no variation of the *T*_m_ with the protein concentration. Both bovine and camel chymosin denatured irreversibly upon heating (Supplementary Fig. S2).

Data processing was performed with the *OriginLab* 7 software. Buffer–buffer scans were subtracted from the protein–buffer scans followed by a baseline subtraction using a cubic function and normalized with the protein concentration to give a profile of excess molar specific heat (*C*_P,m_) as a function of temperature. The melting temperature, *T*_m_, was defined as the temperature at maximum *C*_P,m_.

### 2.9. Crystallization experiments   

Crystallization trials were performed on the most abundant variants of camel chymosin: variants 2 and 3. The crystallization experiments were carried out by the vapour-diffusion method at room temperature. Drops composed of 2 µl protein solution [25 mg ml^−1^ in 50 m*M* bis-tris buffer pH 6.0, 0.05%(*w*/*v*) sodium azide] and 2 µl precipitant were equilibrated against a 1 ml reservoir of precipitant. After 1 d, variant 2 of camel chymosin formed crystals at a protein concentration of 25 mg ml^−1^ using a reservoir consisting of 2 *M* ammonium sulfate, 100 m*M* bis-tris buffer in the pH range 5.1–6.5 (the protein precipitated at pH 4.5). A few large crystals appeared at pH 5.1; they increased in number while decreasing in size as the pH increased. A crystal obtained at pH 5.5 with dimensions of 150 × 100 × 100 µm was used for initial data collection at 100 K. The crystal was cryoprotected in reservoir solution with 0.5 *M* lithium sulfate (Rubinson *et al.*, 2000 ▶). Another data set with improved resolution was collected from a crystal grown under the same conditions but using a protein concentration of 30 mg ml^−1^.

Bovine chymosin was crystallized under conditions similar to those described previously (Gilliland *et al.*, 1990 ▶; Strop *et al.*, 1990 ▶; Newman *et al.*, 1991 ▶). The composition of the reservoir was 100 m*M* NaH_2_PO_4_ pH 5.5, 1.5 *M* NaCl. Each drop was composed of 2 µl reservoir solution and 2 µl bovine chymosin sample at 30 mg ml^−1^ (the commercial product transferred to 50 m*M* NaH_2_PO_4_ pH 6.0). The commercial product contained only one dominant variant; hence, no further separation was deemed necessary. Crystals of diffraction quality appeared after 2 d by seeding with bovine chymosin crystals provided by Chr. Hansen A/S. Crystals of dimensions 100 × 30 × 30 µm were cryoprotected in reservoir solution with 2.5 *M* LiCl (Rubinson *et al.*, 2000 ▶).

### 2.10. Data collection and structure refinement   

Data were collected for both enzymes on the Cassiopeia beamline station I911-2 at MAX-lab, Lund University (Mammen *et al.*, 2002 ▶, 2004 ▶) equipped with a MAR 165 CCD detector at a crystal-to-detector distance of 100 mm. Data reduction and determination of the space group and unit-cell parameters were carried out with the *XDS* software package (Kabsch, 2010 ▶). The crystals of bovine chymosin belonged to space group* I*222, as reported previously, but diffracted to a higher resolution (1.8 Å) than the previously published structures (Gilliland *et al.*, 1990 ▶; Strop *et al.*, 1990 ▶; Newman *et al.*, 1991 ▶). The crystals of camel chymosin belonged to space group *P*2_1_2_1_2_1_. Statistics are summarized in Table 1 ▶. The *CCP*4 suite (Winn *et al.*, 2011 ▶) was used to convert the data into a file format for molecular replacement and to flag 5% of the reflections for calculation of the free *R* factor.

The *PHENIX* software suite (Adams *et al.*, 2010 ▶) was used to solve the structure of camel chymosin by the molecular-replacement method using a trimmed version of bovine chymosin (PDB entry 1cms; Gilliland *et al.*, 1990 ▶; loops and the N-terminus removed) as a search model. The *PHENIX* software suite (Adams *et al.*, 2010 ▶) was used for refinement and the molecular-graphics application *Coot* (Emsley *et al.*, 2010 ▶) was employed for manual building of the model. It was not possible to trace the first ten residues of the N-terminus. In order to investigate this, another data set was collected from a crystal grown from a freshly prepared sample. Although the resolution was improved from 1.9 to 1.6 Å, the N-terminus was also absent in the electron density based on these data. N-­terminal analysis and mass spectrometry showed that the polypeptide chain lacked the first three residues and had the same degree of glycosylation as the original sample. An *N*-­acetylglucosamine (NAG), the first residue in the sugar chain, could be modelled at Asn100. Water molecules were fitted manually in both structures. Higher residual density at the surface of the protein was modelled as ten sulfate ions that could be refined with *B* factors similar to those of the adjacent protein residues. Residual density in the active site was modelled as a glycerol molecule. The side chain of Cys283 was fitted in two different conformations: one corresponding to the disulfide bridge to Cys250 and the other with a disrupted bridge presumably caused by radiation damage during data collection. Dual conformations were also introduced for the side chains of Val32, Ser226 and Val317.

The same refinement procedure was used for the structure of bovine chymosin. Eight of the peaks in the residual density that displayed high density were introduced as chloride ions, the *B* factors of which refined to values that matched those of the protein.

The geometry and local environment of the models were validated with the program *WHAT IF* (Vriend, 1990 ▶). The electron densities of the outliers in the Ramachandran plot are well defined, apart from the loop residues Gln162 in both structures, Gln280 in bovine chymosin and Ser164 in camel chymosin.

Table 2 ▶ contains a summary of the refinement and model statistics.

### 2.11. Electrostatic calculations   

The pI and surface charges of bovine and camel chymosin were calculated with the *Adaptive Poisson-Boltzmann Solver* (*APBS*) software (Baker *et al.*, 2001 ▶), the *PDB*2*PQR* software (Dolinsky *et al.*, 2004 ▶, 2007 ▶) and *PROPKA* 1.0 (Li *et al.*, 2005 ▶). The structural models used were those obtained from the refinement of the two structures, including only structural water molecules and encompassing the full sequence for bovine chymosin and residues 11–323 for camel chymosin. The calculations were carried out for the protein under conditions commonly used in the manufacture of cheese: pH 6.65 with a concentration of free calcium ions of approximately 3 m*M* and a total ionic strength of approximately 80 m*M* (Fox & McSweeney, 1998 ▶) represented by 74 m*M* NaCl.

## 3. Results   

### 3.1. Separation and characterization of variants   

The separation showed that the camel chymosin produced by *A. niger* fermentation contained six different variants of the camel enzyme, as shown in Fig. 1 ▶. They will be referred to as camel chymosin variants 1–6 based on their elution order (as determined by their hydrophobicity). The elution profile of the endoglycosidase-treated sample differs distinctly from the profile of the untreated sample, showing that the variants differ in their glycosylation patterns (Fig. 1 ▶). The six variants also displayed different behaviour on an isoelectric focusing gel (results not shown), supporting their distinct differences. Fig. 1 ▶ shows that in commercial camel chymosin variants 2 and 3 form the major components, while variants 1 and 6 are only present in minor amounts. A similar chromatographic separation of commercial bovine chymosin (Supplementary Fig. S1) showed that it is comprised of two variants. The more abundant variant is unglycosylated, while the other variant is glycosylated at Asn291.

N-terminal analysis, mass spectrometry, differential scanning calorimetry, identification of glycosylation sites and the milk-clotting activity assay were used to characterize the different variants and the commercial camel and bovine chymosin products. The results of these investigations are summarized in Table 3 ▶.

The sequence of camel chymosin suggested two possible N-­glycosylation sites: a more favoured one at Asn100 and a less favoured one at Asn291; according to this, the six variants can be classified into three groups according to their degree of glycosylation. Based on the mass-spectrometric (MS) characterization, variants 1 and 2 are found to be doubly glycosyl­ated, variants 3 and 4 to be singly glycosylated and variants 5 and 6 to be unglycosylated. MS/MS data (not shown) confirmed glycosylation at Asn100 of variant 2. It was identified to be high mannose, with a core structure containing at least 13 mannose residues. The glycosylation site at Asn291 had similar characteristics but contained at least 23 mannose residues. The six variants were also characterized through N-­terminal sequencing. Apart from variant 1, which lacks the first three residues, the other variants had an intact N-­terminus. These results are supported by traditional and off-­line MS/MS analysis (data not shown). Based on these experiments, we conclude that variant 2 possesses the full sequence and is doubly glycosylated at Asn100 and Asn291. The crystals obtained by crystallization of variant 2 contained a protein lacking the first three residues of the N-terminus. It is likely that this has been caused by autocatalysis, as variant 2 had been subjected to an additional separation to remove traces of variant 1 and the expression system did not contain additional peptidases. The double glycosyl­ation of the protein in the crystals was confirmed by MS (Supplementary Fig. S11). Variants 3 and 4 are glycosylated at Asn100 and variants 5 and 6 are unglycosyl­ated. The difference between variants 3 and 4 can be explained by differences in the glycosylation at Asn100. However, it was not possible to identify the cause of the differences in the elution profiles and the masses of the two unglycosylated variants 5 and 6. The degree of glycosylation appears to have an impact on the thermal stability of the variants. The singly glycosylated variant possesses the highest melting point (334.4 K). Both the doubly glycosylated and the unglycosylated variants of camel chymosin have lower melting points; however, they are all significantly higher than the *T*_m_ that was measured for bovine chymosin.

The commercial camel chymosin product has almost twice the milk-clotting activity when compared with the bovine chymosin product. In addition to this, the six variants of camel chymosin show significant variation in their milk-clotting activities (Table 3 ▶). The single and unglycosylated variants (3–6) are those with the highest activity. It is noteworthy that it is the doubly glycosylated variant 1 of camel chymosin that lacks the first three residues which displays the lowest activity.

### 3.2. Structures of bovine and camel chymosin   

**Figure 2:**
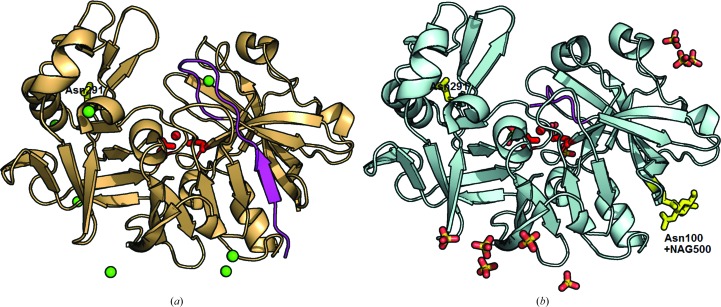
Structures of bovine chymosin (*a*) and camel chymosin variant 2 (*b*). The active-site residues and activated water molecule are shown in red and the N-­terminal residues up to and including Tyr16 are shown in magenta. The experimentally verified glycosylation sites and *N*-acetylglucosamine are shown in yellow and the chloride ions are shown as green spheres; stick models are used for sulfate ions and glycerol.

The crystal structures of bovine and camel chymosin are illustrated in Fig. 2 ▶. In this and all subsequent illustrations, bovine chymosin is shown in light orange and camel chymosin in pale cyan. The rigid cores of the camel and bovine chymosin structures superimpose well, with a root-mean-square deviation (r.m.s.d.) of 0.63 Å (1457 atoms). The structure of bovine chymosin does not differ from the three structures determined previously (Gilliland *et al.*, 1990 ▶; Strop *et al.*, 1990 ▶; Newman *et al.*, 1991 ▶) and can be superimposed with the earlier structures with an r.m.s.d. of 0.39 ± 0.02 Å (comparing 1733 atoms on average). The structural models of the two enzymes contain a significant number of anions close to the positively charged residues at the surface. This is likely to be an effect of the relatively high concentration of salts in the crystallization medium (1.5 *M* NaCl for the bovine enzyme and 2 *M* ammonium sulfate for the camel enzyme). The electron density of the associated cations was comparable to the density of water molecules; even if one considers the differences in the interactions of a cation and a water molecule it was not possible to distinguish any bound cations in the structures of camel and bovine chymosin.

The doubly glycosylated variant 2 was used for the crystallization of camel chymosin. The experimentally verified glycosylation sites of the two structures are marked in yellow in Fig. 2 ▶. Asn100 is located in a β-strand and Asn291 is located in a loop. The structural comparison of bovine and camel chymosin revealed no conformational differences between the two structures in the parts of the structure that carry the two glycosylation sites.

The structural model of camel chymosin contains an NAG residue covalently bound to Asn100 and this does not appear to influence the atomic displacement parameters, which are similar to the values for bovine chymosin. The loop carrying the other glycosylation site at Asn291 is in a flexible part of the molecule as judged from the values of the atomic displacement parameters of the backbone atoms, which are around 40 Å^2^ in both structures, which is significantly above the average value for the protein (Table 2 ▶). The higher mobility of the loop containing Asn291 could explain why it was not possible to identify any carbohydrate bound to Asn291 in the electron density, even though mass spectrometry of the crystals had shown that camel chymosin was glycosylated at both sites. The structural similarity between unglycosylated bovine chymosin and doubly glycosylated camel chymosin leads us to conclude that the post-translational modifications introducing glycosyl­ation do not influence the overall fold of the polypeptide chain of chymosin.

**Figure 3:**
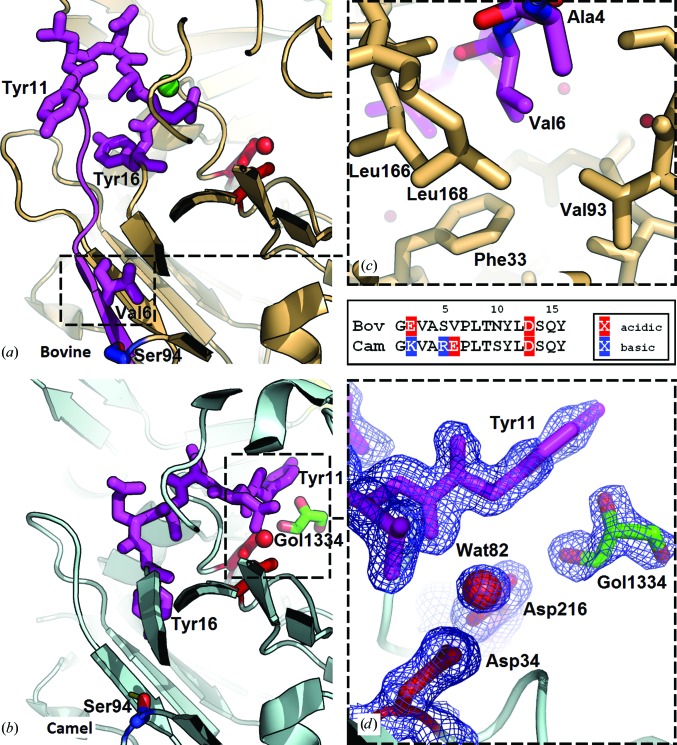
Illustration of the differences in the conformation of the N-terminus of bovine chymosin (*a*) and camel chymosin (*b*) in relation to the central β-sheet. The view is from the N-terminal domain towards the C-­terminal domain. The active-site residues and the activated water molecule are shown in red and the N-­terminal residues up to and including Tyr16 are shown in magenta. The side chain of Ser94 is shown in red/blue and the hydrogen bond formed to the β-sheet in camel chymosin is marked in yellow. The chloride ion in bovine chymosin is illustrated as a green sphere and the glycerol (Gol1334) in camel chymosin is shown in stick representation. (*c*) Enlargement of the environment of Val6 (within 5 Å) in bovine chymosin, where Phe33, Val93, Leu166, Leu168 and solvent interact with the side chain. The sequence and charge of residues 1–16 in camel and bovine chymosin at pH 6.65 are shown in the box, noting that camel chymosin lacks residues 1–3. (*d*) The electron density in the active site of camel chymosin corresponding to the two active-site Asp residues, the activated water molecule, Tyr11 and glycerol (Gol1334). The electron density is traced at the 1.0σ level.

One of the most significant differences between the two structures is in their N-termini (Fig. 3 ▶). In both the present structure of bovine chymosin and those determined by Gilliland *et al.* (1990 ▶), Strop *et al.* (1990 ▶), Newman *et al.* (1991 ▶) and Groves *et al.* (1998 ▶), the N-terminus is visible and forms one of the strands in an antiparallel β-sheet as in other aspartic peptidases such as porcine pepsin (Cooper *et al.*, 1990 ▶; Sielecki *et al.*, 1990 ▶). Residues 5 and 6 are Ser and Val in the bovine enzyme and are Arg and Glu in the camel enzyme. In bovine chymosin the β-strand is formed by residues 4–6, with Ala4 and Val6 forming hydrogen bonds to Leu168 and Leu166, respectively. Replacement of Val6 by Glu would be energetically very unfavourable in the hydrophobic environment of the β-sheet. The differences in the N-termini of the two structures are illustrated in Fig. 3 ▶, in which the residues up to residue 16 are coloured magenta. The first ten residues are not visible in camel chymosin. Despite the fact that the two enzymes have an identical sequence from residues 11 to 16 they adopt completely different conformations, as shown in Fig. 3 ▶. The engagement of residues 4–6 in the β-sheet in bovine chymosin keeps the N-terminal loop in a conformation in which it forms part of the binding cleft; in camel chymosin the same residues point towards the active site. The location of Tyr11 (Fig. 3 ▶) illustrates well the significant differences in conformation between camel and bovine chymosin. In camel chymosin it blocks a major part of the binding cleft and must undergo a conformational change upon binding of substrate. It should be emphasized that the solvent region close to Tyr11 in camel chymosin is sufficiently spacious to be able to accommodate residues 4–10 and does not provide any steric hindrance to conformational changes of residues 10–16. The lack of hydrogen-bond partners for the β-strand formed by residues Leu166–Leu168 is compensated in camel chymosin by a slight displacement of the loop formed by residues 93–96, which enables the side chain of Ser94 to form a hydrogen bond to the carbonyl group of Leu166, thus connecting the β-strand to the loop 93–96.

To elucidate the state of the N-terminus, freshly grown crystals of camel chymosin were used for data collection, resulting in higher resolution. N-terminal analysis, MS and activity measurements on resuspended crystals revealed that the protein lacked the first three residues and had decreased activity, like variant 1.

### 3.3. Surface charge   

**Figure 4:**
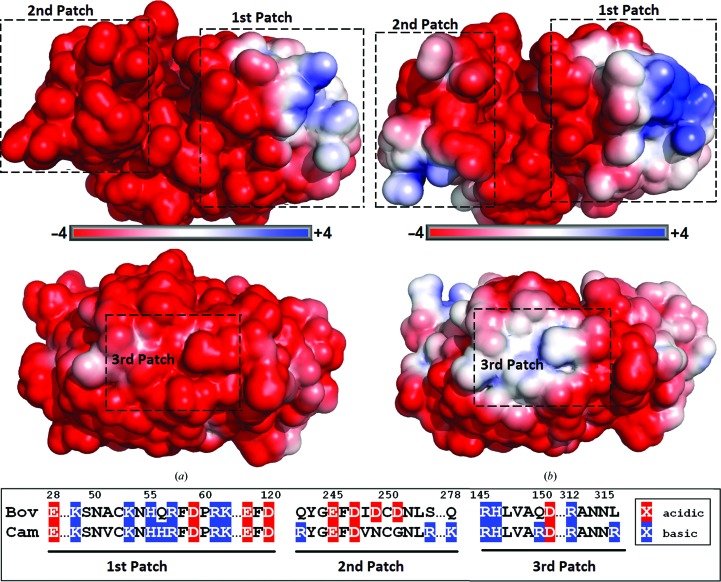
Electrostatic surface plots of bovine (*a*) and camel (*b*) chymosin oriented with the C-terminal domain to the left and the N-terminal domain to the right, looking into the binding cleft (top) and rotated 180° around the horizontal direction (bottom). The scales indicate the charge in *k*_B_ *T*/e_c_. The box shows the sequence of the charged patches; their corresponding positions on the surface are marked in (*a*) and (*b*).

The surface charges of the two enzymes were calculated at pH 6.65, which is the physiological pH of milk (Fox & McSweeney, 1998 ▶). The results are displayed in Fig. 4 ▶. The overall charge of bovine chymosin is −15 e_c_, which is lower than the overall charge for camel chymosin, −9 e_c_, as expected from their pI values: 4.8 for bovine chymosin and 5.4 for camel chymosin (unpublished results from Chr. Hansen A/S). However, the real difference is larger considering that the model of the camel enzyme lacked the first ten residues, which carry a net charge of +1 compared with −1 for the same residues in bovine chymosin (Supplementary Fig. S4). Previous studies of bovine chymosin have identified a positively charged patch on the N-terminal domain and a negatively charged patch on the C-terminal domain adjacent to the substrate-binding cleft; these patches were suggested to influence the interaction with κ-casein (Gilliland *et al.*, 1990 ▶; Newman *et al.*, 1991 ▶). The positively charged patch (roughly corresponding to the first patch in Fig. 4 ▶) is larger in camel chymosin owing to the replacement of a Gln by a His at position 56. The negatively charged patch is less negative in camel chymosin owing to the replacement of Asp249 and Asp251 by Asn249 and Gly251 (part of the second patch). In addition, camel chymosin possesses a small positive patch on the C-terminal domain comprised of residues Arg242, Arg254 and Lys278 (part of the second patch); the corresponding residues in the bovine enzyme are hydrophilic but neutral. The replacement of Gln150 and Leu316 in bovine chymosin by Arg150 and Arg316 in camel chymosin introduces an additional third positive patch on the surface of camel chymosin that contributes to the significant differences in surface charge between camel and bovine chymosin.

## 4. Discussion   

The different degrees of glycosyl­ation observed for the variants of camel chymosin exert only a very small effect on the thermal stability, as shown in Table 3 ▶. The singly glycosylated variants have slightly higher melting points than the doubly and unglycosylated variants. This suggests that glycosylation at Asn100, the more favoured site, with bound NAG is an integral part of a structure with higher molecular mass. Bovine chymosin has a slightly lower (2 K) melting point than the camel enzyme. Even taking the lack of glycosylation into account this is a surprising result, as the disordered N-terminus of camel chymosin could be interpreted as a partial unfolding of the structure, as suggested from studies of other aspartic peptidases (Lin *et al.*, 2000 ▶; Tanaka & Yada, 2001 ▶). The disordered N-terminus leads to the net loss of one hydrogen bond, which could suggest that entropic differences contribute to the slightly higher melting point of camel chymosin relative to bovine chymosin.

The casein micelles in milk are the natural substrate of chymosin. The complex composition and structure of this very large substrate make it particularly challenging to relate differences in the catalytic activity measured as the milk-clotting activity to the structural differences between camel and bovine chymosin. Among the factors that could be expected to affect the enzymatic action of chymosin on casein micelles are the mutual attraction and positioning of the overall negatively charged chymosin in a favourable position that will allow binding to the overall negatively charged C-­terminal part of κ-casein in the active site and the subsequent cleavage of the substrate at the scissile bond *Phe105-Met106*. These points will be considered in the analysis of the structural differences between camel and bovine chymosin.

### 4.1. Impact of glycosylation   

The additional glycosylation site at Asn100 in camel chymosin accounts for the larger number of variants found. The characterization of the variants, summarized in Table 3 ▶, suggests a correlation between the milk-clotting activity and the location of the glycosylation site relative to the binding site. The glycosylation site at Asn291 is located close to the entrance to the substrate-binding cleft (Fig. 2 ▶) and could impair substrate binding and thus explain the significantly lower activities of the doubly glycosylated camel chymosin variants 1 and 2, which are approximately 25 and 60%, respectively, of that of variant 5 (see Table 3 ▶). The other glycosylation site at Asn100 is located far away from the binding cleft (Fig. 2 ▶), and variants 3 and 4, which are glycosyl­ated only at this site, have the same milk-clotting activity as the unglycosylated variant 5. The drop in the measured activity of variant 3 was caused by the presence of a small amount of variant 2, as seen in Fig. 1 ▶ and Supplementary Figs. S6 and S7.

### 4.2. Surface charge   

Bovine κ-casein is a relatively small milk protein comprising 169 residues; it has been shown that residues 97–112 play a role in the interaction with chymosin and that the last part of the C-terminus is disordered, which is consistent with the extensive O-glycosylation of the C-terminus (Fox & McSweeney, 1998 ▶). Several residues in the sequence of κ-­casein close to the *Phe105-Met106* bond are positively charged (Supplementary Fig. S4) under cheese-making conditions (pH 6.65) and fit well with the overall negative charge of bovine and camel chymosin. On the other hand, the remaining part of the C-terminus of κ-casein is negatively charged owing to the presence of several Asp and Glu residues (Fox & McSweeney, 1998 ▶) and might therefore be expected to be repelled by the negatively charged chymosin, an effect that will be less pronounced for camel chymosin since it possesses a smaller negative charge. However, as can be seen from the electrostatic surface plots in Fig. 4 ▶, both bovine and camel chymosin contain positively charged areas (the first and second patches, the latter of which is only present in camel chymosin). These are located just outside the substrate-binding cleft, which may aid in orienting the enzyme and its subsequent association with the negatively charged C-terminal part of κ-casein. The chloride ions in bovine chymosin and the sulfate ions in camel chymosin are located adjacent to these patches, demonstrating the impact of the positively charged patches of the structures. The negatively charged C-­terminus of κ-casein will be repelled by the negative patches and attracted by the positive patches. These interactions can facilitate the binding of the C-terminus in the active site. Both the electrostatic surface maps in Fig. 4 ▶ and the locations of the anions in the structures illustrated in Fig. 2 ▶ revealed the existence of an additional positive patch on the surface of camel chymosin (the third patch), which could be of importance for the attraction of the negatively charged C-terminus of κ-­casein. This is in agreement with a previous suggestion that the positively charged residues in the stretch 48–62 of bovine chymosin could aid in stabilization of the enzyme–substrate complex (Gilliland *et al.*, 1990 ▶). The additional positive charges of camel chymosin are therefore likely to contribute to its improved milk-clotting properties relative to bovine chymosin.

### 4.3. N-terminus   

The most significant structural difference between camel chymosin and bovine chymosin is observed in their secondary-structural elements. A prominent secondary structure in bovine chymosin is the central six-stranded antiparallel β-­sheet at the ‘bottom’ of the active site, which links and stabilizes the relative orientation of the two domains in the structure. The N-terminus of bovine chymosin forms one of the strands in this sheet. The disordered N-terminus of camel chymosin implies that this structure lacks one of the strands, which leads to a breakdown of the pseudo-twofold symmetry that relates the two domains of the chymosin structure (Newman *et al.*, 1991 ▶). It has been shown that the two domains of chymosin move relative to each other upon inhibitor binding (Šali *et al.*, 1992 ▶; Groves *et al.*, 1998 ▶), and the relative domain movement within camel chymosin must be influenced by the breakdown of symmetry.

A comparison of the sequences of camel chymosin and bovine chymosin (Supplementary Fig. S4) shows why it would not be possible for the N-terminus of the camel enzyme to adopt the same conformation as the N-terminus of bovine chymosin. The presence of the large charged Arg5 and Glu6 residues (Ser5 and Val6 in bovine chymosin) adjacent to each other precludes the participation of the N-terminus in the β-­sheet, as one of them must be buried in the hydrophobic core of the protein. In bovine chymosin the β-strand is formed by residues 4–6, with Ala4 and Val6 hydrogen bonded to Leu168 and Leu166, respectively. The replacement of Val6 by Glu would be energetically unfavourable in the hydrophobic environment.

Arg5 and Glu6 in camel chymosin make it unfavourable for the N-terminus to adopt a well defined conformation embedded in the structure. This could explain why it points into the solvent, giving rise to a less compact crystal packing of camel chymosin compared with the bovine enzyme. Among the other significant differences between the sequences of the N-termini of camel and bovine chymosin is the overall positive charge of the N-terminus of the camel enzyme, which is partly owing to the substitution of a Glu in position 2 of bovine chymosin by a Lys in camel chymosin. The decrease in activity associated with the lack of the first three residues in the sequence shows the significance of the positively charged and disordered N-terminus. One could even envision that the positively charged disordered N-terminus of camel chymosin acts as a bait for the negatively charged disordered C-terminus of κ-casein, facilitating binding of the substrate.

### 4.4. Substrate binding   

Significant experimental challenges are associated with determining the structure of chymosin with a bound inhibitor, but in 1998 Groves and coworkers succeeded in preparing and determining the structure of bovine chymosin with a bound inhibitor (Groves *et al.*, 1998 ▶). This structure revealed that a rigid-body movement of the two domains relative to each other took place upon binding, similar to that observed for other aspartic peptidases (Šali *et al.*, 1992 ▶). In addition, Tyr77 undergoes a conformational change in bovine chymosin that enables the substrate to enter the binding cleft, as described previously by Gilliland *et al.* (1990 ▶) and Newman *et al.* (1991 ▶). Andreeva *et al.* (1992 ▶) have previously identified residues that may influence the mobility of Tyr77, and as they are virtually identical in bovine and camel chymosin we conclude that the improved milk-clotting properties of camel chymosin are unlikely to be related to a change in the mobility of Tyr77.

Chen *et al.* (1992 ▶) studied the structure of the pepstatin–pepsin complex and showed a correlation between the domain movement and the size of the bound inhibitor (Groves *et al.*, 1998 ▶). In view of the differences in secondary structure between bovine and camel chymosin described above, one would expect camel chymosin to be more flexible, with a greater ability to accommodate substrates and inhibitors of different natures.

**Figure 5:**
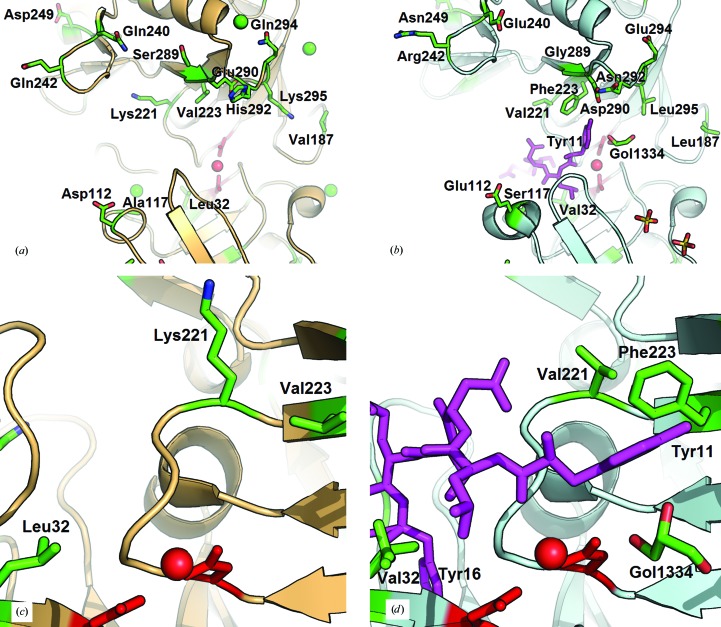
The substrate-binding clefts of bovine (*a*, *c*) and camel (*b*, *d*) chymosin. The active-site residues and the activated water molecule are illustrated in red. Residues 11–16 of camel chymosin are shown in magenta. Glycerol (Gol1334) and the residues that differ between bovine and camel chymosin are shown in green (Table 4 ▶). (*a*) and (*b*) illustrate the loops that form the entrance to the binding site. (*c*) Subsite S1 with Leu32 and subsites S2 and S4 with Lys221 and Val223. (*d*) The corresponding subsites in camel chymosin with Val32 (both conformations) and with Val221 and Phe223, respectively.

The hydrogen-bonding network that positions the Asp34 and Asp216 catalytic machinery is identical in bovine and camel chymosin, and it seems unlikely that it would be affected by structural variations of the substrate-binding cleft. The inhibitor structure determined by Groves *et al.* (1998 ▶) mapped out subsites S4 to S1′ of bovine chymosin. This information has enabled us to identify the residues delineating the substrate-binding site that differ between bovine and camel chymosin and that may affect the specific binding of the substrate (Table 4 ▶). The S1 and S1′ subsites are relatively large and hydrophobic, as observed for most aspartic peptidases (Kay & Dunn, 1992 ▶), and seem to be well suited to accommodate the side chains of *Phe105* and *Met106* of κ-casein. The additional electron density that was found in the active site is located in subsite S1′ and was modelled as glycerol. Although the exact nature of the bound molecule is unknown and it does not appear to make any hydrogen bonds to the surrounding protein residues, it shows that subsite S1′ possesses a propensity for binding smaller molecules. In a mutational study of chymosin in which Val111 (which forms part of S1 and S3) was mutated to Phe (the corresponding residue in pepsin) the enzyme showed a significant decrease in activity related to the decreased substrate affinity (Strop *et al.*, 1990 ▶). In this respect, it is interesting to note that Leu32 in bovine chymosin is replaced by Val in the camel enzyme, which creates a slightly larger S1 subsite. A minor difference in subsite S3 (binding *Leu103*) is the replacement of Ala117 in the bovine enzyme by Ser in camel chymosin. More remarkable differences between the residues delineating the substrate-binding cleft of bovine and camel chymosin are observed for binding sites S2 and S4 (which bind *Ser104* and *His102*, respectively), in which the substrate may interact with Lys221 and Val223 in bovine chymosin, which are replaced by Val221 and Phe223 in camel chymosin (Fig. 5 ▶). These changes influence the shape, volume and charge of the substrate-binding site. Recent molecular-modelling studies have also indicated that these residues play a role in substrate binding. Palmer *et al.* (2010 ▶) studied bovine chymosin and suggested that Lys221 interacts with the uncharged *His102* of κ-casein and that Val223 does not take part in substrate interactions at pH 6.65 (the normal milk-clotting conditions). In similar modelling investigations of camel chymosin, Sørensen *et al.* (2011 ▶) proposed that the large aromatic Phe223 interacts with *Ser104* and *His102.* However, structure determinations of other aspartic peptidases with bound inhibitors (Epps *et al.*, 1990 ▶; Hong *et al.*, 2000 ▶), which have revealed a great ability to adjust the binding site to the different inhibitors, do not lend support to the role of *His102* proposed from the modelling studies. The observed malleability of substrate binding in aspartic peptidases suggests that the substrate may show variations in binding to subsites S4 to S2 between bovine and camel chymosin. The finer details of the substrate binding can therefore only be revealed by knowledge of the structure of chymosin with bound substrate (inhibitor). Our structural comparison of bovine and camel chymosin suggests that differences in surface charge can facilitate the binding of camel chymosin to κ-casein. Unfortunately, computational methods for proteins have not yet reached a state that enables the modelling of the interactions of an entire casein micelle with chymosin.

Following the promising industrial applications of camel chymosin first reported by Kappeler *et al.* (2006 ▶), we have aimed to identify the structural origins of the improved clotting of bovine milk by camel chymosin compared with bovine chymosin. Taking the complexity of the natural substrate casein into consideration, we find that the improved milk-clotting activity of camel chymosin can be attributed to variations in the surface charge that facilitate the association between camel chymosin and the casein micelles. In addition, the increased mobility of camel chymosin, combined with significant differences in the residues that delineate the substrate-binding cleft, contribute to improved substrate binding by camel chymosin.

## Supplementary Material

PDB reference: bovine chymosin, 4aa8

PDB reference: camel chymosin, 4aa9

Supporting information file. DOI: 10.1107/S0907444913003260/wd5199sup1.pdf

## Figures and Tables

**Table 1:** Data-collection and processing statistics Values in parentheses are for the highest resolution shell.

	Bovine chymosin	Camel chymosin
Space group	*I*222	*P*2_1_2_1_2_1_
Unit-cell parameters ()		
*a*	71.6	53.3
*b*	79.2	66.1
*c*	113.2	133.7
Mosaicity ()	0.220	0.101
Wavelength ()	1.04	1.04
Resolution range ()	30.01.80 (1.901.80)	30.01.60 (1.701.60)
Total reflections	243754	539439
Unique reflections	30113	62765
Multiplicity	8.1 (8.0)	8.6 (8.6)
*R*_merge_ †	0.079 (0.498)	0.036 (0.321)
Completeness (%)	99.7 (99.0)	99.2 (97.8)
*I*/(*I*)	19 (5)	31 (6)

† *R*_merge_ = 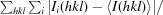 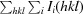.

**Table 2:** Structure-refinement and validation statistics

	Bovine chymosin	Camel chymosin
Resolution range ()	26.61.80	24.81.60
*R*_free_ †	0.215	0.208
*R*_work_ ‡	0.177	0.186
Reflections (total)	30027	62748
Reflections (*R*_free_ test set)	1520	3182
Solvent content (%)	46	58
Molecules in the asymmetric unit	1	1
No. of atoms	2890	2968
No. of amino-acid residues	323	312
No. of carbohydrates	0	1
No. of anions	8	10
No. of solvent molecules	370	442
Average *B* factor (^2^)		
Overall	23.4	24.4
Amino acids	22.2	21.6
Anions	26.1	52.0
Carbohydrates		48.1
Ligand		41.4
Water molecules	31.7	35.8
R.m.s.d. from ideal		
Bond lengths ()	0.008	0.007
Bond angles ()	1.032	1.061
Ramachandran outliers	Leu12, Ser94, Gln162, Gln189, Gln280	Tyr134, Gln162, Ser164, Gln189

† *R*_work_ = 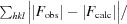 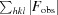, where *F*_calc_ is the structure factor calculated from the model and *F*_obs_ is the structure factor of the observed reflections used for model building.

‡ *R*_free_ = 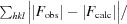 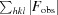, where *F*_calc_ is the structure factor calculated from the model and *F*_obs_ is the structure factor of the observed reflections retained from model building (the *R*_free_ test set).

**Table 3:** Properties of the separated variants of camel chymosin For comparison, commercial bovine and camel chymosin have milk-clotting activities of 223 and 462IMCUmg^1^, respectively (data from Chr. Hansen A/S). Their melting points were determined to be 330.8 and 333.8K, respectively.

Variant	1	2	3	4	5	6
Sequence†	4323	1323	1323	1323	1323	1323
Mass spectrometry						
Peak‡ (kDa)	40.2	40.3	37.7	37.7	35.6	35.8
Range§ (kDa)	39.042.0 (49.051.5)	39.542.0	36.538.5	36.539.0 (35.036.0)		
Glycosylation	Asn100, Asn291	Asn100, Asn291	Asn100	Asn100		
Activity (IMCUmg^1^)	123 2	289 1	396 1	467 1	474 5	426 8
*T*_m_ ¶ (K)	333.0	333.6	334.4	334.4	332.5	332.2

† The full sequence of mature bovine and camel chymosin consists of residues 1323.

‡ The average mass of the peaks in the spectra.

§ For the heterogenously glycosylated camel chymosin variants 14 the mass range is given as the full width at half maximum (the spectra have been deposited as Supplementary Material). Values in parentheses represent minor peaks in the spectra.

¶ The melting point, *T*_m_, is defined as the temperature at maximum molar specific heat, *C*_P,m_.

**Table 4:** Differences in the amino-acid residues delineating the substrate-binding cleft in bovine and camel chymosin subsites

Residue	Bovine	Camel	Subsite
32	Leu	Val	S1
112	Asp	Glu	
117	Ala	Ser	S3
187	Val	Leu	
221	Lys	Val	S4
223	Val	Phe	S2 + S4
240	Gln	Glu	
242	Gln	Arg	S9 + S10
249	Asp	Asn	
289	Ser	Gly	
290	Glu	Asp	
292	His	Asn	
294	Gln	Glu	S1 + S3
295	Lys	Leu	

## Footnotes

1 Supplementary material has been deposited in the IUCr electronic archive (Reference: WD5199). Services for accessing this material are described at the back of the journal.
