# Supplementary material for: Camel and bovine chymosin: the relationship between their structures and cheese-making properties
Source: Acta Crystallogr D Biol Crystallogr. 2013 Apr 19;69(Pt 5):901–13. doi: 10.1107/S0907444913003260 (PMC3640475; doi:10.1107/S0907444913003260)
Supplement: Supplementary file 1 [file d-69-00901-sup1.pdf]

Camel and Bovine Chymosin: the Relationship Between their Structures and Cheese Making Properties

Supplementary Material

Jesper Langholm Jensen[ab], Anne Mølgaard[a], Jens-Christian Navarro Poulsen[a], Marianne Kirsten Harboe[b], Jens Bæk Simonsen[c1], Andrea Maria Lorentzen[d], Karin Hjernø[d], Johannes M. van den Brink[b], Karsten Bruun Qvist[b] and Sine Larsen[a]*

[a]Department of Chemistry, University of Copenhagen, Denmark, [b]Chr. Hansen A/S, Bøge Allé 10-12, Hørsholm, DK-2970, Denmark, [c]Nanobioscience, Department of Basic Sciences and Environment, University of Copenhagen, Denmark, and [d]Institute of Biochemistry and Molecular Biology, University of Southern Denmark, Denmark

Correspondence email: sine@chem.ku.dk

Chromatography

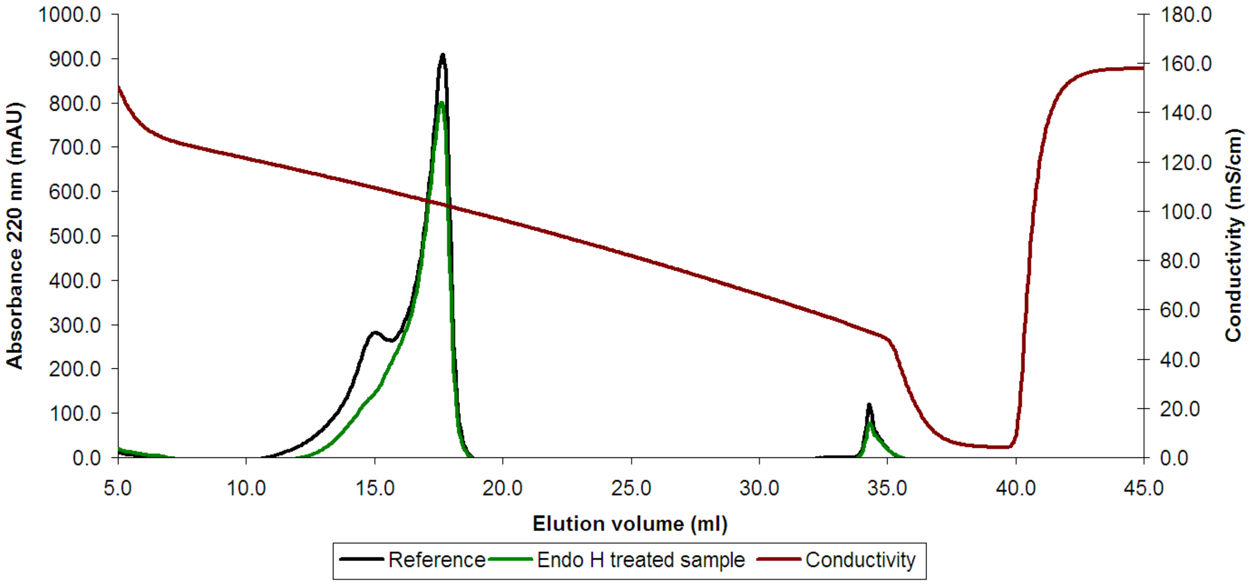

Figure S1. Representative chromatogram showing the FPLC separation of the commercial product of bovine chymosin against a salt gradient on a hydrophobic column. The black chromatogram shows the separated variants of the product. The green chromatogram shows the separation of the product pretreated with endoglycosidase H.

Differential Scannning Calorimetry

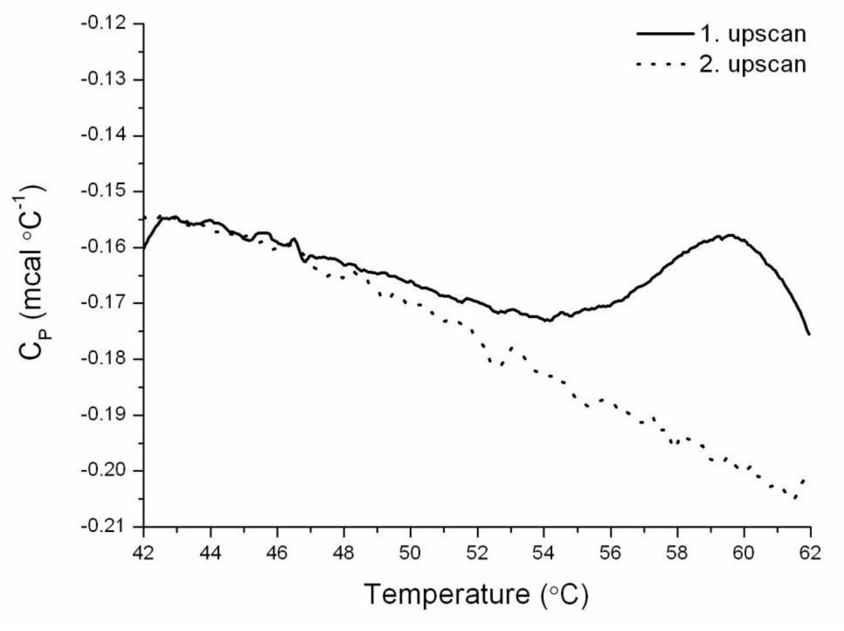

Figure S2. Raw DSC data for camel chymosin variant 5. The variants of bovine and camel chymosin denatured irreversibly upon heating. An endothermic contribution from denaturation can be seen in the first upscan after sample injection. This is absent in the second upscan.

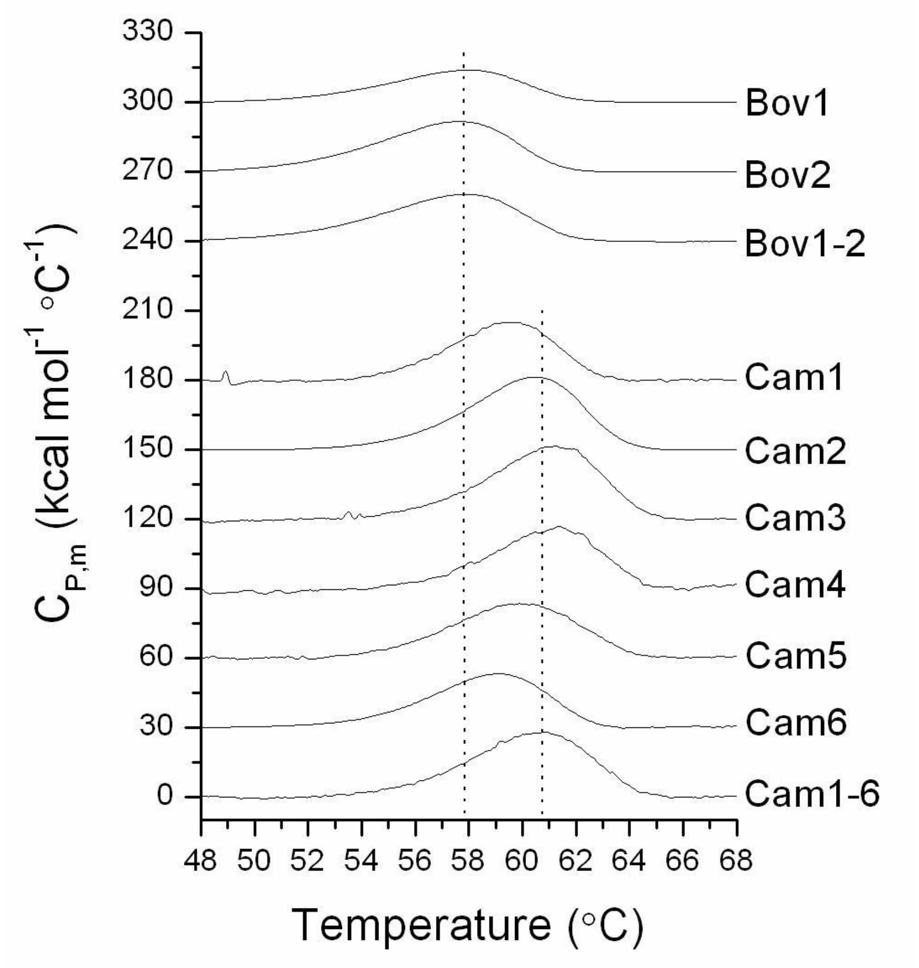

Figure S3. Melting curves of the variants of bovine (Bov1+2) and camel chymosin (Cam1+2+3+4+5+6), and the commercial products (Bov1-2+Cam1-6). For clarity, the curves are displaced in steps of 30,000 kcal / (mol · °C) along the vertical axis with respect to Cam1-6. The dashed lines indicate the melting point of the commercial products

Sequences

Pro-sequence

```
            10p       20p       30p       40p
Bov AEITRIPLYKGKSLRKALKEHGLLEDFLQKQQYGISSKYSGF
Cam SGITRIPLHKGKTLRKALKERGLLEDFLQRQQYAVSSKYSSL
```

Mature sequence

```
            10        20        30        40        50        60
Bov GEVASVPLTNYLDSQYFGKIYLGTPPQEFTVLFDTGSSDFWVPSIYCKSNACKNHQRFDP
Cam GKVAREPLTSYLDSQYFGKIYIGTPPQEFTVVFDTGSSDLWVPSIYCKSNVCKNHHRFDP
     *  **   *           *         *       *          *    *

            70        80        90       100       110       120
Bov RKSSTFQNLGKPLSIHYGTGSMQGILGYDTVTVSNIVDIQQTVGLSTQEPGDVFTYAEFD
Cam RKSSTFRNLGKPLSIHYGTGSMEGFLGYDTVTVSNIVDPNQTVGLSTEQPGEVFTYSEFD
          *               * *             **       **  *    *

           130       140       150       160       170       180
Bov GILGMAYPSLASEYSIPVFDNMMNRHLVAQDLFSVYMDRNGQESMLTLGAIDPSYYTGSL
Cam GILGLAYPSLASEYSVPVFDNMMDRHLVARDLFSVYMDRNGQGSMLTLGAIDPSYYTGSL
        *          *       *     *            *

           190       200       210       220       230       240
Bov HWVPVTVQQYWQFTVDSVTISGVVVACEGGCQAILDTGTSKLVGPSSDILNIQQAIGATQ
Cam HWVPVTLQQYWQFTVDSVTINGVAVACVGGCQAILDTGTSVLFGPSSDILKIQMAIGATE
          *             *  *   *            * *       *  *     *

           250       260       270       280       290       300
Bov NQYGEFDIDCDNLSYMPTVVFEINGKMYPLTPSAYTSQDQGFCTSGFQSENHSQKWILGD
Cam NRYGEFDVNCGNLRSMPTVVFEINGRDYPLSPSAYTSKDQGFCTSGFQGDNNSELWILGD
     *     ** *  **          **   *      *          ** * **

           310       320
Bov VFIREYYSVFDRANNLVGLAKAI
Cam VFIREYYSVFDRANNRVGLAKAI
                   *
```

X acidic	N glycosylated
X basic	* Difference

Bovine κ-casein

```
            10        20        30        40        50        60
Cas QEQNQEQPIRCEKDERFFSDKIAKYIPIQYVLSRYPSYGLNYYQQKPVALINNQFLPYPY
            70        80        90       100     ↓ 110       120
Cas YAKPAAVRSPAQILQWQVLSNTVPAKSCQAQPTTMARHPHPHLSFMAIPPKKNQDKTEIP
           130       140       150       160   ↑
Cas TINTIASGEPTSTPTTEAVESTVATLEDSPEVIESPPEINTVQVTSTAV
```

Figure S4. (top) Alignment of the sequences of the prosegment and mature protein of bovine and camel chymosin based on Uniprot entries P00794 and Q9GK11, respectively. (bottom) The sequence of bovine κ-casein based on Uniprot entry P02668. The *Phe105-Met106* bond cleaved by chymosin is marked with arrowheads.

Mass spectra

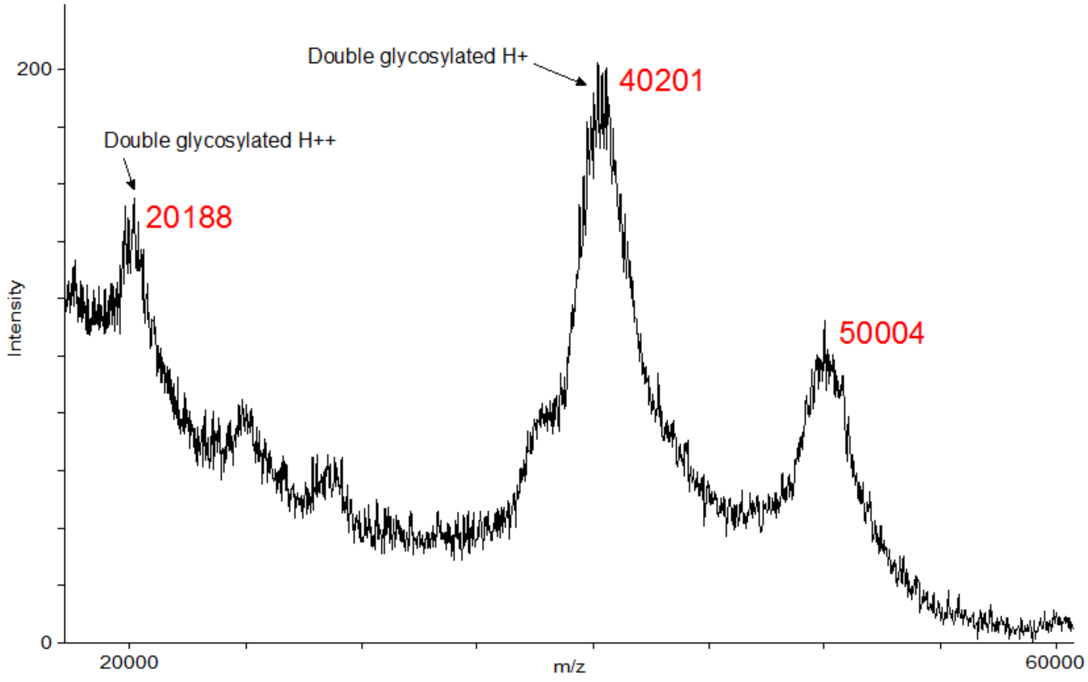

Figure S5. Mass spectra of camel chymosin variant 1.

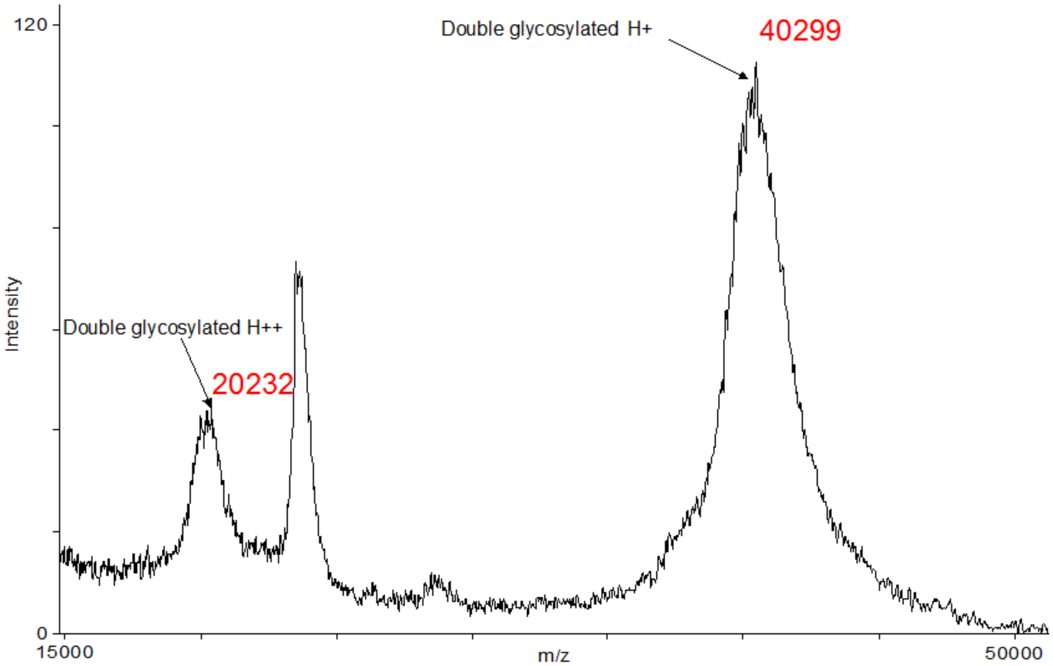

Figure S6. Mass spectra of camel chymosin variant 2.

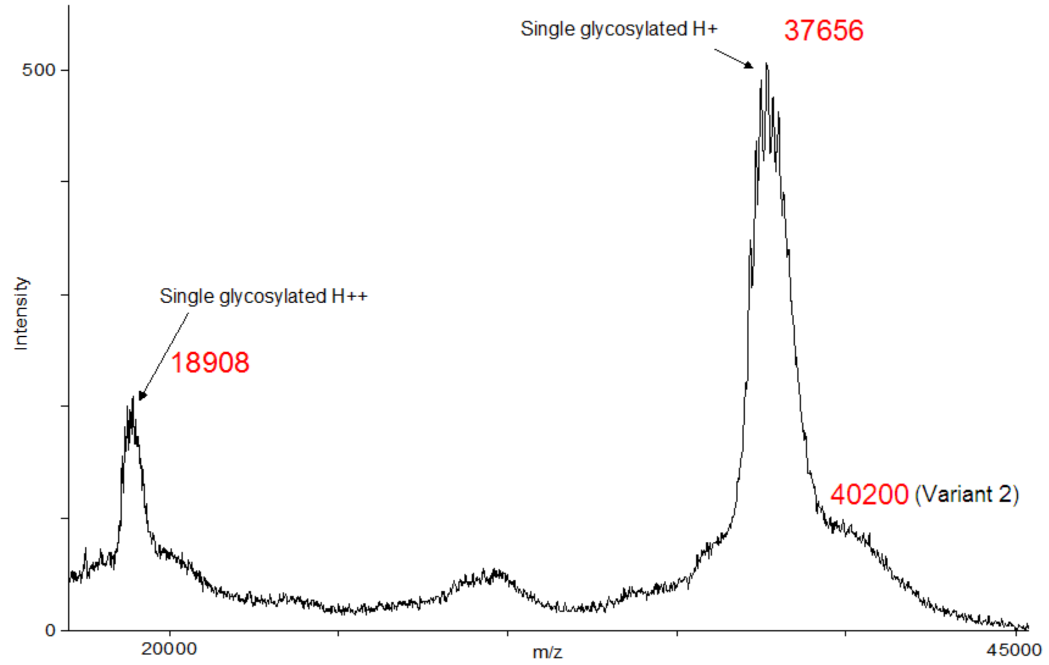

Figure S7. Mass spectra of camel chymosin variant 3.

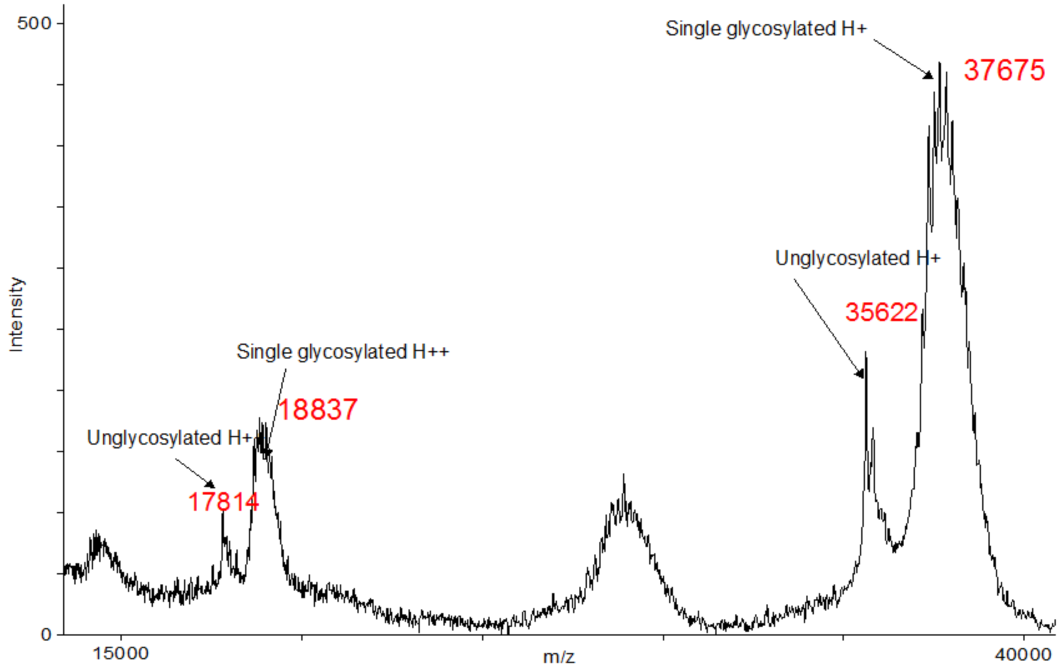

Figure S8. Mass spectra of camel chymosin variant 4.

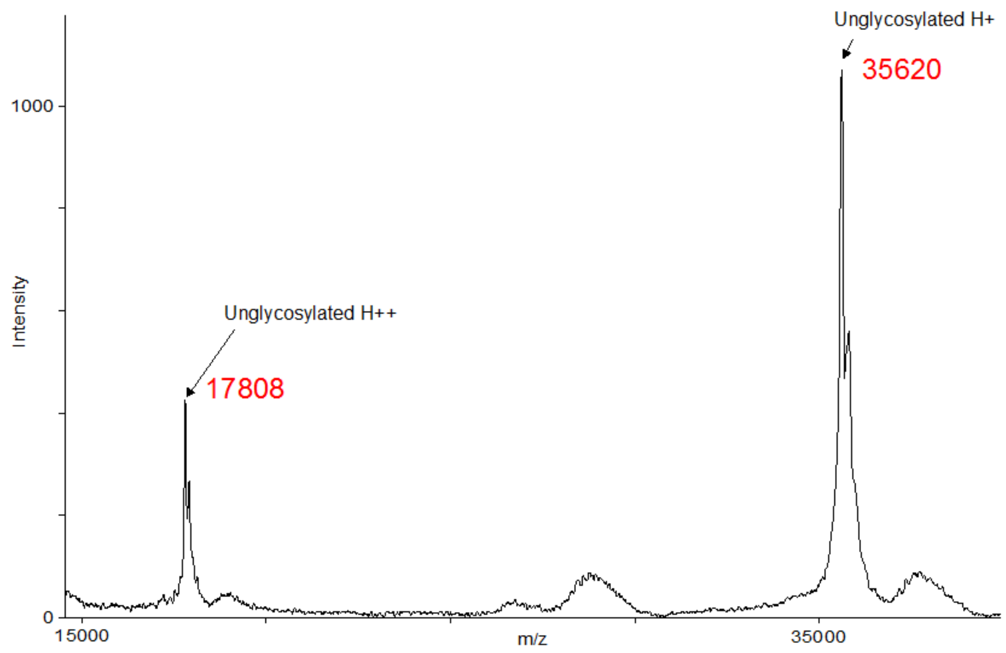

Figure S9. Mass spectra of camel chymosin variant 5.

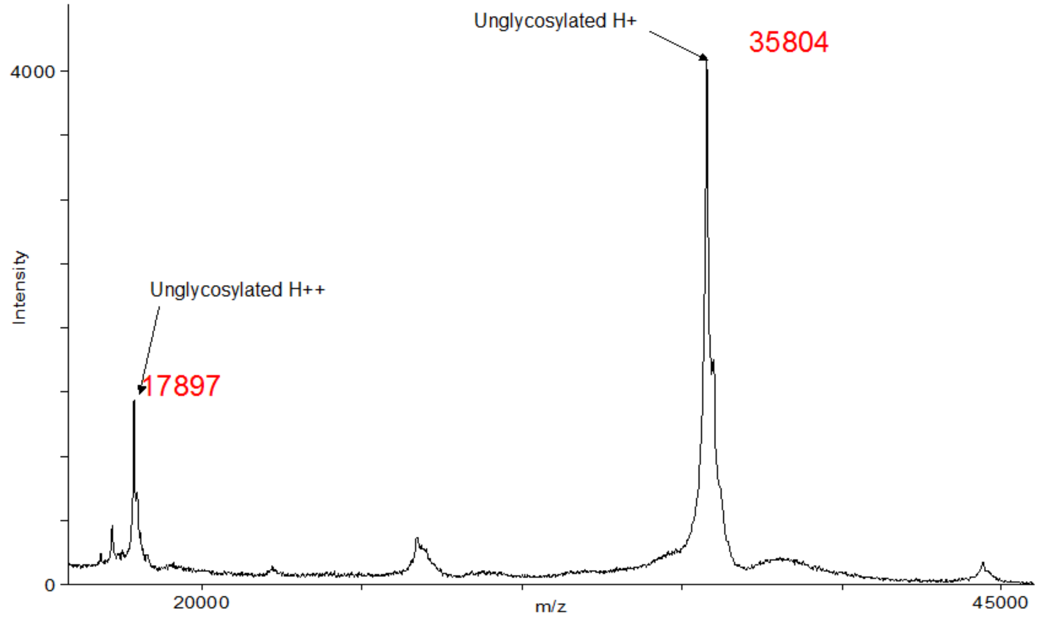

Figure S10. Mass spectra of camel chymosin variant 6.

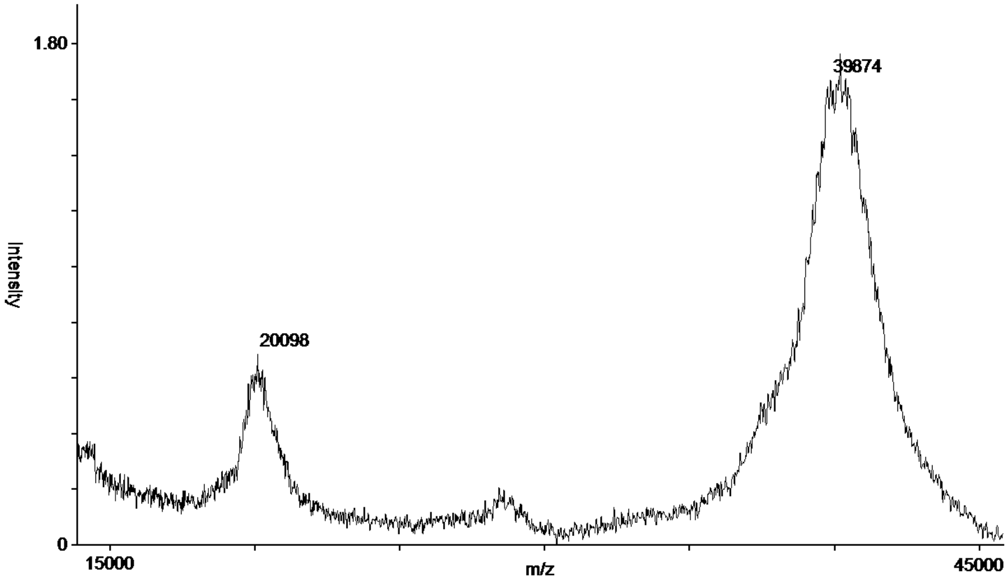

Figure S11. Mass spectra of crystals of camel chymosin variant 2.
